# Development and Verification of Postural Control Assessment Using Deep-Learning-Based Pose Estimators: Towards Clinical Applications

**DOI:** 10.1155/2022/6952999

**Published:** 2022-11-30

**Authors:** Naoto Ienaga, Shuhei Takahata, Kei Terayama, Daiki Enomoto, Hiroyuki Ishihara, Haruka Noda, Hiromichi Hagihara

**Affiliations:** ^1^Faculty of Engineering, Information and Systems, University of Tsukuba, Japan; ^2^Aino University, Osaka, Japan; ^3^Graduate School of Medical Life Science, Yokohama City University, Kanagawa, Japan; ^4^LITALICO Inc., Tokyo, Japan; ^5^Nippon Telegraph and Telephone, Kanagawa, Japan; ^6^Graduate School of Biomedical Sciences, Nagasaki University, Nagasaki, Japan; ^7^International Research Center for Neurointelligence (WPI-IRCN), The University of Tokyo Institutes for Advanced Study, Tokyo, Japan; ^8^Japan Society for the Promotion of Science, Tokyo, Japan

## Abstract

Occupational therapists evaluate various aspects of a client's occupational performance. Among these, postural control is one of the fundamental skills that need assessment. Recently, several methods have been proposed to estimate postural control abilities using deep-learning-based approaches. Such techniques allow for the potential to provide automated, precise, fine-grained quantitative indices simply by evaluating videos of a client engaging in a postural control task. However, the clinical applicability of these assessment tools requires further investigation. In the current study, we compared three deep-learning-based pose estimators to assess their clinical applicability in terms of accuracy of pose estimations and processing speed. In addition, we verified which of the proposed quantitative indices for postural controls best reflected the clinical evaluations of occupational therapists. A framework using deep-learning techniques broadens the possibility of quantifying clients' postural control in a more fine-grained way compared with conventional coarse indices, which can lead to improved occupational therapy practice.

## 1. Introduction

Occupational therapy is provided to a wide range of clients, from young children to elderly adults, to help people develop or regain skills needed for participation in daily activities [1]. To achieve this goal, occupational therapists (OTs) perform a holistic assessment of clients' body functions, activities, participation, personal and environmental factors, and their interrelationships [2]. OTs evaluate patients' abilities, such as motor skills, sensations, perceptions, sociality, and human communication skills through different tasks. In the field of pediatric and school occupational therapy, body functions, such as postural control, provide a fundamental basis for a client's occupational performance including self-care, human communication, and academic learning [3]. A large proportion of people with developmental disorders generally have impaired postural control skills [4–10], although these impairments are sometimes overlooked [11, 12]. Thus, evaluation of postural control is essential for occupational therapy [13].

However, sufficiently sensitive indices are lacking in the existing assessment batteries [14–16] as they tend to use duration, speed, or the number of successful trials within a certain time range, which often leads to a ceiling effect. OTs, therefore, perform clinical qualitative evaluations based on observations, but such detailed descriptions are not reflected in the quantitative measurements. They often use specialized equipment (e.g., body sway machines) to quantify and visualize a client's motor skill performance despite less clinical availability in terms of financial cost [17] or time taken for preparation and/or processing.

**Figure 1:**
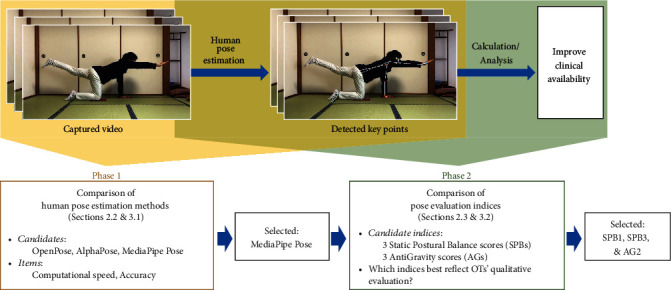
Flowchart of this study.

One solution is to leverage deep-learning or computer vision technologies to develop fine-grained quantitative assessment tools that can be easily used in clinical settings. These new-generation technologies do not require OTs to buy expensive specialized equipment. Furthermore, if well defined, it would provide them with detailed summary indices calculated from just recorded videos. Over the last decade, deep learning has been successfully applied to various fields such as medicine, education, and psychology (e.g., [18–22]), dramatically contributing to both basic and clinical research related to human behavior. Our previous work focused on pediatric occupational therapy [13] aimed to quantify OT's qualitative observational evaluations of a task called “bird dog posture.” This task comes from the Japanese Playful Assessment for Neuropsychological Abilities (JPAN) [16, 23, 24], which is a developmental assessment battery for evaluating children's sensory integration abilities. In this task, participants are instructed to maintain their right (left) arm and left (right) leg as horizontal (or higher) as possible (see Figure 1). The task ends when either the individual's arm or leg touches the floor or 60 seconds passes. Although this task is typically scored by the duration of time, our previous work [13] succeeded in quantifying children's static postural balance score (SPB) and antigravity score (AG) using only a video recorded on a camera. It used a human pose estimation method, OpenPose [25], to detect the keypoints of body parts from a 2D image and calculate these quantitative indices.

Although our previous work [13] made an important first step in utilizing deep-learning techniques in rehabilitation settings, further investigation is necessary to fully evaluate the clinical applicability of the developed assessment tools. The current study addresses two limitations of our previous research, which would apply to other similar existing studies. First, the previous study [13] focused on only one pose estimation algorithm, but there are other alternatives, such as AlphaPose [26] or MediaPipe Pose [27, 28]. A substantial number of methods have been recently proposed, and some of them are unique and have highly accurate estimations. It would be beneficial to compare these algorithms to determine which human pose estimation method is best suited for clinical application. Second, in [13], SPB and AG were intuitively formulated and developed; however, other candidate indices exist. The process of how to combine and calculate keypoints obtained from pose estimators should be further examined to develop indices that better reflect qualitative clinical evaluation.

In the present study, we address these limitations by comparing the accuracy and processing speed of three representative and well-established human pose estimation methods: OpenPose, AlphaPose, and MediaPipe Pose (Figure 1). We then propose and evaluate an automated construction of indices that better reflects OTs' observational qualitative evaluation of a client's postural control abilities. Specifically, we define two additional alternative indices for both SPB and AG. Refinement of these indices is significant, especially when assessing the postural control of older children and adults. These refined methods can provide fine-grained quantitative scores for postural control that can help with the limitations of simply using duration time, such as the ceiling effect resulting from these individuals easily reaching the maximum of 60 seconds.

The structure of this paper is as follows. First, we explain in detail the participants and the datasets in addition to the proposed method in Section 2. Then, we present comparison results (1) of human pose estimation methods for accuracy and calculation time and (2) pose evaluation indices for reflection of OTs' qualitative evaluation in Section 3. Next, we discuss the results and their clinical applicability in Section 4. Finally, an overall conclusion is provided in Section 5. For readers' convenience, Supplementary Table S1 describes all the acronyms and abbreviations used in this article.

## 2. Materials and Methods

### 2.1. Participants and Datasets

Two datasets were used for this study: a child and an adult dataset. The child dataset was identical to our previous study [13], in which 34 typically developing preschoolers (Mean age = 4.7, SD = 1.0, range = 3–6 years old; 14 girls) participated. The adult dataset included unpublished data, in which 23 adults (mean age = 23.8, SD = 4.6, range = 19–35 years old; 11 females) participated. The research was approved by the Research Ethics Committee of Hakuho College (18012), and written informed consent was obtained from each participant's caregiver (child dataset) or the actual participant (adult dataset).

Each dataset consisted of video data where participants performed a postural control assessment called “One Arm and One Leg Balance,” which is one of the subtests of the Japanese Playful Assessment for Neuropsychological Abilities (JPAN) [16, 23, 24]. The JPAN is a developmental assessment battery for evaluating sensory integration abilities for children aged 4 to 10 years old and was developed in Japan based on existing assessment tools, such as Sensory Integration and Praxis Tests [29]. In the One Arm and One Leg Balance task, a participant is asked to maintain what is called “bird dog posture” for a maximum of 60 seconds on each side, in which the opposite arm and leg are lifted from a four-point crawling posture. This task demands an unfamiliar postural balance [30]; therefore, a participant's splinter skills regarding their postural control would be less likely to affect the task performance.

Each video has a mean task performance score where three pediatric OTs evaluated the overall quality of postural control based on their clinical viewpoint using a 7-point Likert scale (7 indicates the most superior performance, see our previous work [13] for more details).

### 2.2. Pose Estimation Methods

As noted, only OpenPose was used in the previous study. In the current study, three open-source human pose estimation methods are compared in terms of estimation accuracy and processing speed. The numbers in parentheses are the versions of the methods used.

- OpenPose (1.7.0) [25] is well-known and popular and is one of the representative bottom-up human pose estimation methods. Bottom-up methods first estimate all positions that seem to be human keypoints from the entire image, then group the keypoints for each person afterward. Since bottom-up methods process all people in the image simultaneously, they have the advantage of inference at high speed, even if the number of people increases. OpenPose can detect a total of 137 keypoints (70 for the face, 25 for the body, and 21 for each hand)
- AlphaPose (0.5.0) [26] is one of the representative top-down human pose estimation methods. The top-down methods first detect each person in the input image, then the keypoints are estimated for each detected person. In general, top-down methods can estimate keypoints with higher accuracy than bottom-up methods. AlphaPose also can track people and detect a total of 136 keypoints (68 for the face, 26 for the body, and 21 for each hand)
- MediaPipe Pose (0.8.10) [27, 28] targets a single person, but it runs on a mobile CPU in near-realtime. This is expected to be suitable for clinical application. In addition, 3D keypoint estimation is possible from a single RGB image. Unlike OpenPose and AlphaPose, Mediapipe can only detect keypoints of a single person. MediaPipe detects 33 keypoints for the body (they also provide many other solutions, such as MediaPipe Hands and MediaPipe Face Mesh)

When applying AlphaPose to a high-resolution video, it did not work properly. Therefore, when processing video, the video size was reduced in all methods (when the height of the video was 1000 px (pixel) or more, the height and width were one-third, and when the height was less than 1000 px, the height and width was halved). Moreover, following the previous study, the input videos were rotated 90° to turn the participants' heads upward. Note that the heights of raw videos in the dataset ranged from 720 px to 1080 px, and the widths ranged from 1280 px to 1920 px. The FPS (frames per second) was 30. When processing the image, only rotation was applied. Hyperparameters were not adjusted, and the default values were used for the three methods.

A comparison of the three methods revealed that MediaPipe Pose had better speed and accuracy than the others, so it was selected as the main method for the current study (see Section 3.1 for details). The keypoint positions estimated by MediaPipe Pose were denoised using the following method. The keypoints with visibility (see Section 3.1) less than 0.5 were considered outliers. Moreover, we calculated the exponentially weighted moving standard deviation (EWMSD), *σ*, and considered a keypoint position to be an outlier when the difference between the exponentially weighted moving average (EMA) of the keypoint positions was more than *σ* apart. The window lengths of the EWMSD and EMA are equal to the FPS of the input video. This procedure was repeated five times to remove outliers. Removed outliers were interpolated linearly.

### 2.3. Pose Evaluation Indices

Pose evaluation indices were divided into six groups: three SPBs and three AGs. SPBs aim to quantify how stable the body is, and AGs attempt to quantify how much gravity can be resisted. SPB1 and AG1 were designed based on the previous work [13] with minor modifications.

To explain SPB1 and 2, we introduced moving distance {*D*_*n*−1_^*k*^} as follows:

(1) $$\begin{array}{c} \left\{D_{n-1}^{k}\right\}=\frac{d_{1,2}^{k}}{t},\cdots ,\frac{d_{i,i+1}^{k}}{t},\cdots ,\frac{d_{n-1,n}^{k}}{t}. \end{array}$$

*d*_*i*−1,*i*_^*k*^ indicates the moving distance of the 2D image between two consecutive frames *i* − 1, *i* of keypoint *k*. *k* is a variable that represents the four keypoints of the extended limbs: 1 (elbow), 2 (wrist), 3 (knee), and 4 (ankle). *n* is the number of video frames, and *t* represents trunk length. *t* is the average value of distance between the shoulder and hip in all frames of the video. *t* could contribute to influences due to differences in participants' height and in the distance between the camera and the participants. SPB1 is the average value of {*D*_*n*−1_^*k*^} over the frames and keypoints.

(2) $$\begin{array}{c} \text{SPB}1=\frac{1}{4}\overset{4}{\underset{k=1}{\sum }}\left(\frac{1}{n-1}\overset{n-1}{\underset{i=1}{\sum }}D_{i}^{k}\right). \end{array}$$

SPB2 is similar to SPB1, but focuses on larger changes. The four keypoints' {*D*_*n*−1_^*k*^} are summed for each element to get the sequence {*D*_*n*−1_}. The maximum value of {*D*_*n*−1_} for SPB2 is as follows:

(3) $$\begin{array}{c} \left\{D_{n-1}\right\}=\overset{4}{\underset{k=1}{\sum }}D_{1}^{k},\cdots ,\overset{4}{\underset{k=1}{\sum }}D_{i}^{k},\cdots ,\overset{4}{\underset{k=1}{\sum }}D_{n-1}^{k},\\ \text{SPB}2=\max\left(\left\{D_{n-1}\right\}\right). \end{array}$$

Suppose the max function returns the maximum value in the sequence.

Unlike SPB1 and SPB2, SPB3 is not based on the keypoints' moving distance. The area of the convex hull drawn by *k*'s movement is calculated. SPB3 is the total area of four convex hulls divided by *n*.

**Figure 2:**
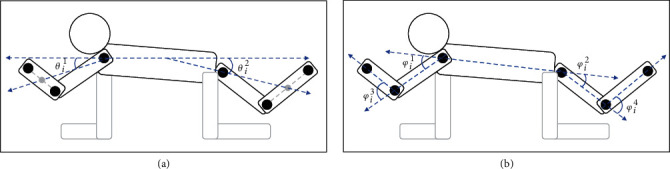
A schematic diagram of the angles at frame *i* used in the calculation of AGs. *θ*s are used for AG1 (a), and *φ*s are used for AG2 and AG3 (b). All angles range from 0 to 180 degrees.

Figure 2 explains the angles used in calculating the AGs. The angle *θ*_*i*_^1^ (*θ*_*i*_^2^) at frame *i* is formed by the vector connecting the shoulder (hip) and the midpoint of the elbow (knee) and wrist (ankle), and the horizontal vector of the image. AG1 is calculated as follows:

(4) $$\begin{array}{c} \text{AG}1=\frac{1}{n}\overset{n}{\underset{i=1}{\sum }}\left(\theta_{i}^{1}+\theta_{i}^{2}\right). \end{array}$$

However, AG1 has three problems. First, raising the arm (foot) above the shoulder (hip) results in a negative evaluation. This should not receive a poorer evaluated as it means resisting gravity. The second is that AG might not be evaluated properly when the arm or leg is bent. The third is that AG1 assumes that the video is kept horizontally. In AG2 and 3, four angles *φ*_*i*_^1^, *φ*_*i*_^2^, *φ*_*i*_^3^, *φ*_*i*_^4^ are calculated for each frame *i*. *φ*_*i*_^1^ (*φ*_*i*_^2^) is formed by the vector connecting the shoulder and hip and the vector connecting the elbow (knee) and shoulder (hip). *φ*_*i*_^1^ (*φ*_*i*_^2^) is 0 when the elbow (knee) is above the shoulder (hip). *φ*_*i*_^3^ (*φ*_*i*_^4^) is formed by the vector connecting the elbow (knee) and shoulder (hip) and the vector connecting the wrist (ankle) and elbow (knee). AG2 and AG3 are calculated as follows:

(5) $$\begin{array}{c} \left\{\Phi_{n}\right\}=\overset{4}{\underset{k=1}{\sum }}\varphi_{1}^{k},\cdots ,\overset{4}{\underset{k=1}{\sum }}\varphi_{i}^{k},\cdots ,\overset{4}{\underset{k=1}{\sum }}\varphi_{n}^{k},\\ \text{AG}2=\frac{1}{n}\overset{n}{\underset{i=1}{\sum }}\Phi_{i},\\ \text{AG}3=\max\left(\left\{\Phi_{n}\right\}\right). \end{array}$$

All programs in this study were implemented on Google Colaboratory.

## 3. Results

### 3.1. Comparison of Human Pose Estimation Methods

**Figure 3:**
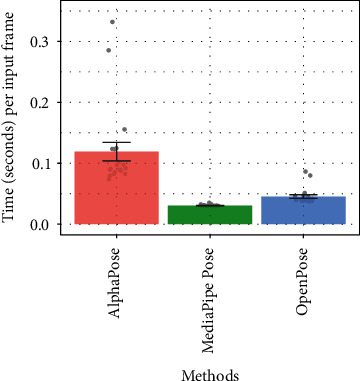
Mean processing time of each pose estimation method. Note: error bars indicate standard error.

First, we assessed the three target human pose estimation methods (i.e., OpenPose, AlphaPose, and MediaPipe Pose) in terms of processing speed and accuracy. Processing speed was considered to practically reflect computational complexity because, generally speaking, a more complex method takes a longer time to process the input data. For processing speed, we used 20 videos of 5 children and 5 adults (each participant performed the task on the right and left sides). We calculated the total time taken to load the video, estimate the postural landmarks, and complete the output for keypoint positions for each method. We then divided the processing time by the number of input video frames. The mean time per frame was equivalent for OpenPose (*M* = 0.045 s, SD = 0.013) and MediaPipe (*M* = 0.030 s, SD = 0.001), whereas it took approximately three or four times longer in AlphaPose (*M* = 0.119 s, SD = 0.068) than in the other methods (Figure 3).

For accuracy, we randomly extracted 10 frames from each of the videos used for assessing processing speed (10 frames × 10 participants × 2 sides = 200 target frames). For each pose estimation method, to measure estimation error, we calculated the distance (px) between manually annotated and automatically estimated keypoints for the target keypoints of the shoulder, elbow, wrist, hip, knee, and ankle for the extended limbs. Since frame resolutions and the distance between the camera and participant varied across videos, we divided the estimation error by the trunk length *t* that was calculated based on the manual annotation to control for individual differences. AlphaPose and OpenPose produce an estimation of keypoints with corresponding “confidence values” ranging from 0 to 1. The higher value indicates that the keypoint is more likely to be estimated accurately. MediaPipe Pose produces a similar index called “visibility.” We evaluated the acceptance rate of keypoints and the estimation error distance per trunk length while moving the threshold of such confidence or visibility values.

**Figure 4:**
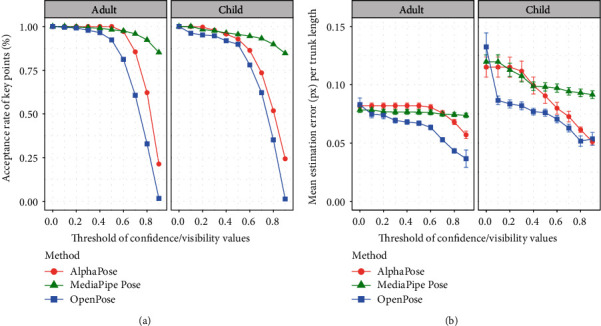
Acceptance rate and estimation error distance of each pose estimation method. Note: error bars indicate standard error.

Acceptance rates dropped in OpenPose and AlphaPose as the thresholds for confidence values were set higher (i.e., more strict exclusion criteria), whereas those in MediaPipe Pose were less affected by the thresholds of visibility (Figure 4(a)). In particular, almost all keypoints were excluded in OpenPose (acceptance rate of 1.3% and 1.6% for the child and adult samples, respectively) with a threshold of 0.9 or greater for confidence values. On the other hand, more than 80% of the samples remained in MediaPipe Pose, even when the threshold of visibility was set at 0.9 (84.7% of the child sample and 85.2% of the adult sample).

The mean estimation error distance per trunk length decreased in OpenPose and AlphaPose as the threshold of confidence values became higher (Figure 4(b)). The extent of this decrease seemed greater in the child sample than in the adult sample, and OpenPose was the most accurate method among the three candidate algorithms. However, the mean estimation error was consistent in MediaPipe Pose regardless of the threshold of visibility values, particularly for the adult sample. For the child sample, the error distance in MediaPipe Pose decreased slowly, especially when the threshold was set above 0.5.

Thus, in AlphaPose and OpenPose, there was a tradeoff between the acceptance rate and accuracy of the keypoints, whereas MediaPipe Pose showed a robust high acceptance rate, although its accuracy was relatively lower than the other two methods when the threshold was high. These results were similar when the measurements were evaluated for each keypoint (see Supplementary Figures S1 and S2). Although the mean estimation error distance in MediaPipe Pose with a threshold of visibility of 0.5 and above ranged from 0.08 to 0.10 (i.e., approximately 10% of the trunk length), these errors may be clinically acceptable for a gross motor assessment. Based on data used in the current study, this length is 20% of the forearm and 15% of the lower thigh length.

Overall, based on the assessment of processing speed and accuracy, we decided to use MediaPipe Pose with a visibility threshold of 0.5 to maximize clinical usability.

### 3.2. Comparison of Pose Evaluation Indices

To select the indices that best predict the clinical evaluation of OTs, linear regression models were constructed using the entire child and adult datasets for each of the SPBs and AGs. We used a backward stepwise selection method with AIC (Akaike's Information Criterion) values. The smaller AIC value indicated a better model in terms of the model's predictability.

For the SPB models, we first created the full model in which SPB1, SPB2, and SPB3 were entered as independent variables. The best model (AIC = 0.14, adjusted *R*^2^ = 0.62) included SPB1 (*β* = −1.88, SE = 0.19, *p* < 0.001) and SPB3 (*β* = 0.77, SE = 0.19, *p* < 0.001), but not SPB2. These selected variables were consistent even when the model fitting was performed for the child and adult datasets separately; however, the direction of the effects differed. The best model using the child dataset (AIC = −22.26, adjusted *R*^2^ = 0.39) showed a similar result to the model using both datasets, where SPB1 had a negative effect on the occupational therapists' clinical evaluation score (*β* = −0.93, SE = 0.21, *p* < 0.001) and SPB3 had a positive effect on it (*β* = 0.27, SE = 0.18, *p* = 0.15). Meanwhile, for the best model using the adult dataset (AIC = −28.7, adjusted *R*^2^ = 0.69), both SPB1 (*β* = −1.42, SE = 0.38, *p* < 0.001) and SPB3 (*β* = −2.39, SE = 1.12, *p* = 0.039) showed negative effects. Thus, it is plausible that the greater the SPB1 value, the lower the clinical evaluation score of the OT.

For the AG models, we also created a full model in which AG1, AG2, and AG3 were included as independent variables. AG1 and AG2, but not AG3, remained in the best model identified using both datasets (AIC = 46.7, adjusted *R*^2^ = 0.43). Both AG1 (*β* = −0.35, SE = 0.25, *p* = 0.16) and AG2 (*β* = −0.74, SE = 0.25, *p* = 0.0036) were negatively associated with the OTs' clinical evaluations. When using the child and adult datasets separately, the best model included only AG2 (best model using the adult dataset: AIC = 4.94, adjusted *R*^2^ = 0.33, AG2's *β* = −1.32, SE = 0.27, *p* < 0.001; best model using the child dataset: AIC = −8.30, adjusted *R*^2^ = 0.24, AG2's *β* = −0.52, SE = 0.11, *p* < 0.001). Therefore, what was consistently confirmed was that a greater AG2 value indicates a lower clinical evaluation score.

Finally, we constructed a model including the selected independent variables with and without duration time, and compared these models with the conventional model including only duration time as an independent variable (Table 1). Regardless of which dataset was used (child and adult, child only, or adult only), the model including the selected variables with duration time was superior to the model including only duration time (ΔAIC = 56.49 for the model using both datasets; ΔAIC = 22.96 for the model using the child dataset; ΔAIC = 38.13 for the model using the adult dataset). For reference, the mean scores of the selected variables for each dataset are shown in Table 2.

## 4. Discussion

The primary objectives of this study were to compare which of the three pose estimators (AlphaPose, MediaPipe Pose, and OpenPose) was the most clinically applicable in terms of accuracy for keypoints and processing speed and to verify which of the proposed indices regarding postural control abilities best reflected clinical evaluations of OTs.

Among the three pose estimators, OpenPose and MediaPipe Pose took relatively less time for processing. Given that OpenPose needed additional time for the first implementation, MediaPipe Pose was the best choice in terms of speed for estimating keypoints for our specific task. When applying AlphaPose to a high-resolution video, reduction of the video resolution was needed. In terms of accuracy, OpenPose outperformed the other two methods at a certain threshold of confidence values, but the acceptance rate of keypoints drastically decreased as the threshold got higher. AlphaPose showed a similar trend, but the accuracy of keypoints was relatively worse. On the contrary, MediaPipe Pose was not sensitive to the threshold visibility values, and the acceptance rate remained high. Its mean estimation error remained at around 0.08 or 0.10 px with a threshold of 0.5. This error length was approximately 10% of the trunk length, around 20% of the forearm, and 15% of the lower thigh length. We considered these errors to be clinically acceptable for a gross motor assessment because, for instance, the keypoint estimation of a client's wrist is still much closer to the correct position of the wrist than other keypoints, such as the elbow. Therefore, given the processing speed, ease of implementation (i.e., it can work on CPU), and acceptable error distances, we decided to use MediaPipe Pose for our study. However, if a researcher or therapist attempts to detect each keypoint more precisely (e.g., fine motor skills [14, 15]), it might be recommended to use other methods, such as OpenPose.

When comparing the static postural balance scores, SPB1 was consistently related to the clinical evaluation score of OTs. This suggests that the greater the SPB1 value, the lower the OTs' score. The SPB1 definition was the same as our previous work [13], so this intuitive index would be a plausible quantification of the qualitative aspect of postural control skills. On the other hand, SPB3 which was defined by a convex hull also remained in the final model, although the direction of its regression coefficient was incongruent depending on the model. Using the adult dataset, the regression coefficients for SPB1 and SPB3 were negative, allowing for the interpretation that the smaller the area of the convex hull, the better the evaluation of postural control. However, the regression coefficient for SPB3 sometimes turned out to be positive, especially when using the child dataset. This might be due to the fact that the area of the convex hull became smaller when the hand or foot touched the floor for a relatively short time because it became like a long and thin line. Alternatively, this counterintuitive result may have happened due to multicollinearity between SPB1 and SPB3. Although the convex hull is used as a quantitative index of postural control, such as body sway (e.g., [31]), this index may not be appropriate for the type of task where limb movements are like a pendulum. Based on the study results, SPB1 should be considered as an index of static balance in a clinical setting.

For the model comparison of the antigravity scores, AG2 was consistently included in the final model. When the client's elbow (or knee) flexed, the wrist (or ankle) could be located at a high position, leading to a seemingly well-maintained antigravity posture. In fact, AG1 showed a better score for this kind of posture in some cases. However, AG2 was defined in a way that the score would be worse for such a “tricky” posture. This discernibility of AG2 would be more reasonable for clinical settings.

Regardless of the type of dataset, the selected variables contributed to lowering AIC. This suggests that the OTs' qualitative evaluation of postural control could be more appropriately and quantitatively reflected when SPB and AG indices are taken into account than when duration time alone is used. This tendency was observed in the model using the adult dataset where duration time showed a ceiling effect. Of the 23 participants, 22 succeeded in maintaining their posture for a maximum of 60 seconds. Although simple duration time or the number of successful trials has been conventionally used as quantitative indices (e.g., [16]), other fine-grained quantifications, such as SPB and AG may be more meaningful for more appropriately and precisely evaluating a client's postural control abilities. Deep-learning techniques enable therapists to perform such quantifications simply by recording a client's posture using an ordinary video camera.

To apply the methodology proposed in the current study for clinical settings, we provided Python codes to calculate SPB and AG scores from video input, as well as mean scores for each index for children and adults for reference. When taking a video of a client's posture, we recommend that (1) the background and the client's clothes should be easily distinguishable, and (2) the camera should be fixed at the same height as the client's trunk and kept in a horizontal position, rather than hand-held. These points are important for accurate pose estimation.

For future work, we raise three points here. First, the application of deep-learning-based pose estimators for other tasks should be thoroughly investigated. For instance, there are other subtasks used for evaluating postural controls in existing assessment batteries [14–16]. Furthermore, it would be better if a therapist could obtain a quantitative measurement by taking a video of a client engaging in daily activity in a natural setting. Second, the provision of a standardized score for SPB and AG and their developmental trends would be helpful as in other indices [32]. Notably, a study with a large sample size would be required for this. Third, deep-learning-based methods have not been perfected, so if the algorithm is improved, the indices of postural control would also be improved as a result. In fact, there were some cases of failure in posture estimation in the current study. Finally, explainable artificial intelligence (XAI) is attracting attention to clarify and understand the reasons for decisions made by AI and machine learning models [33, 34]. In the future, by training a prediction model of the postural control performance on large-scale data and using XAI methods, it may be possible to extract sensory indicators such as SPB and AG, which are considered interpretable features.

## 5. Conclusions

This study compared three pose estimators (AlphaPose, MediaPipe Pose, and OpenPose) to determine which was the most clinically applicable in terms of accuracy for keypoints and processing speed. Also, we verified which of the proposed indices regarding postural control abilities best reflected clinical evaluations of OTs. The framework using deep-learning techniques expands the possibility of quantifying clients' postural control in a more fine-grained way compared with conventional coarse indices. The development of automated quantification shown in the current study can be used to improve occupational therapy practice.

## Figures and Tables

**Table 1:** Regression modeling results.

Model	Predictor	*β* (SE)	*p* value	AIC	Adjusted *R*^2^
Adult and child datasets (combined)					
Selected variables with duration time	SPB1	-0.71 (0.14)	<0.001	-112.10	0.86
	SPB3	0.42 (0.12)	<0.001		
	AG1	-0.22 (0.12)	0.081		
	AG2	-0.15 (0.13)	0.26		
	Duration time	0.97 (0.08)	<0.001		

Selected variables without duration time	SPB1	-1.47 (0.20)	<0.001	-14.85	0.68
	SPB3	0.60 (0.18)	<0.001		
	AG1	-0.28 (0.19)	0.14		
	AG2	-0.21 (0.20)	0.30		
Duration time only	Duration time	1.41 (0.07)	<0.001	-55.61	0.77

Child dataset only					
Selected variables with duration time	SPB1	-0.27 (0.12)	0.032	-111.02	0.84
	SPB3	0.10 (0.09)	0.28		
	AG2	-0.23 (0.06)	<0.001		
	Duration time	0.82 (0.07)	<0.001		

Selected variables without duration time	SPB1	-0.76 (0.21)	<0.001	-28.57	0.45
	SPB3	0.21 (0.17)	0.22		
	AG2	-0.30 (0.10)	0.0051		
Duration time only	Duration time	1.00 (0.07)	<0.001	-88.07	0.77

Adult dataset only					
Selected variables with duration time	SPB1	-1.11 (0.32)	0.0013	-54.73	0.83
	SPB3	1.61 (1.16)	0.17		
	AG2	-0.69 (0.18)	<0.001		
	Duration time	1.51 (0.28)	<0.001		

Selected variables without duration time	SPB1	-1.00 (0.42)	0.021	-31.51	0.71
	SPB3	-2.75 (1.09)	0.016		
	AG2	-0.47 (0.22)	0.039		
Duration time only	Duration time	1.89 (0.24)	<0.001	-16.60	0.58

**Table 2:** Means and standard deviations for the selected variables.

	SPB1	SPB3	AG1	AG2	Duration time
Adult *M* (SD)	0.00217 (0.00218)	0.0177 (0.0261)	26.1 (14.92)	74.3 (32.4)	57.5 (11.8)
Child *M* (SD)	0.00703 (0.00447)	0.1387 (0.1853)	47.7 (20.89)	131.6 (58.7)	29.2 (21.4)

Note: *M*: mean; SD: standard deviation.

## Data Availability

The codes for calculating each proposed index (SPB and AG) from a video file (mp4) are available in the GitHub repository (https://github.com/decobocollabo/Postural-Control-Assessment). We did not make the raw video datasets used in this study publicly available owing to ethics issues. Requests to access the anonymized datasets such as calculated SPB for each participant should be directed to the corresponding author.

## Supplementary Materials

Supplementary Materials Figure S1: Acceptance rate of each pose estimation method for each keypoint. Figure S2: Estimation error distance of each pose estimation method for each keypoint. Table S1: List of abbreviations.
