# Supplementary material for: Development and Verification of Postural Control Assessment Using Deep-Learning-Based Pose Estimators: Towards Clinical Applications
Source: Occup Ther Int. 2022 Nov 30;2022:6952999. doi: 10.1155/2022/6952999 (PMC9729024; doi:10.1155/2022/6952999)
Supplement: Supplementary Materials — Figure S1: Acceptance rate of each pose estimation method for each keypoint. Figure S2: Estimation error distance of each pose estimation method for each keypoint. Table S1: List of abbreviations. [file 6952999.f1.pdf]

Supplementary materials

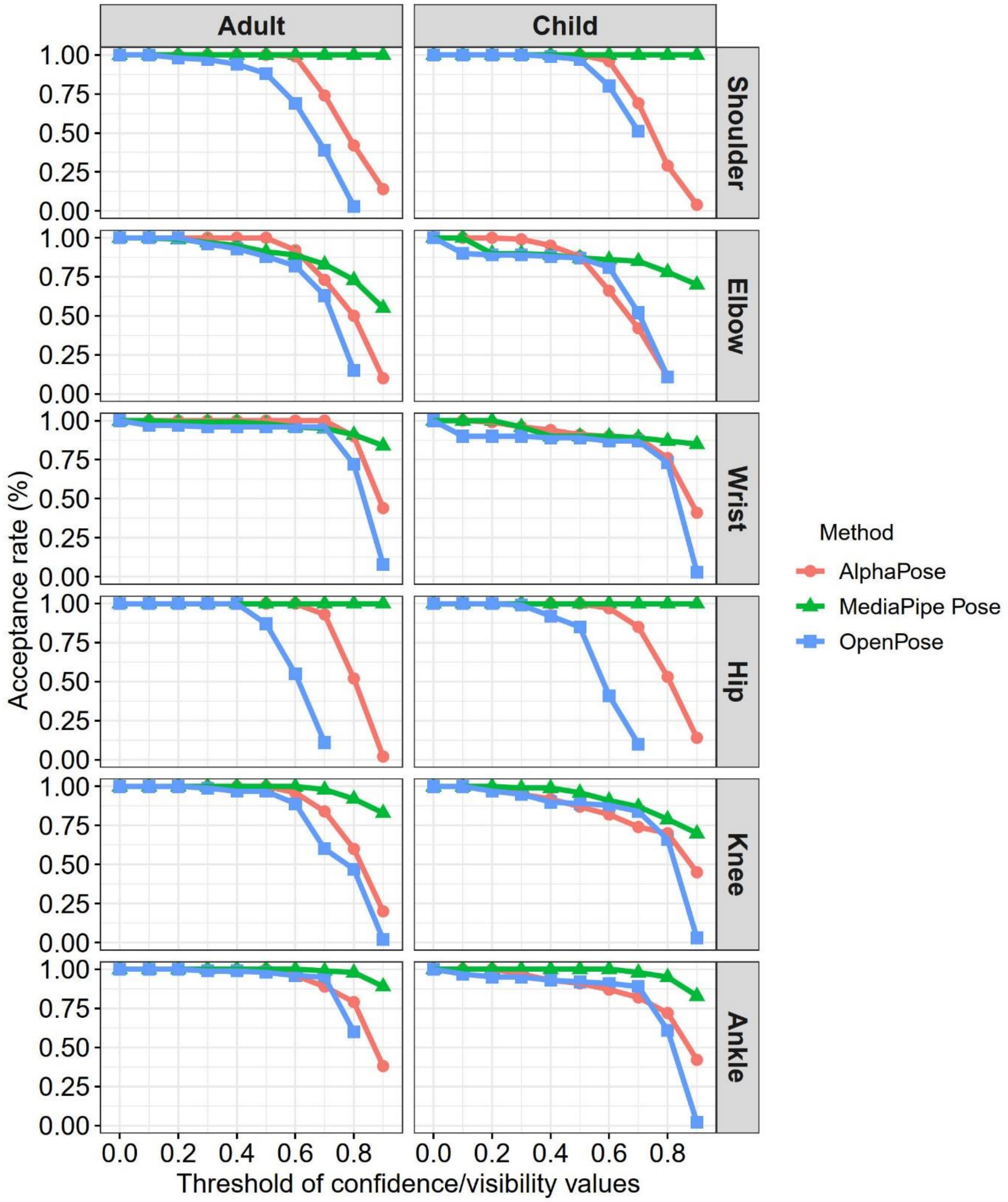

Figure S1. Acceptance rate of each pose estimation method for each keypoint.
Note. Error bars indicate standard error.

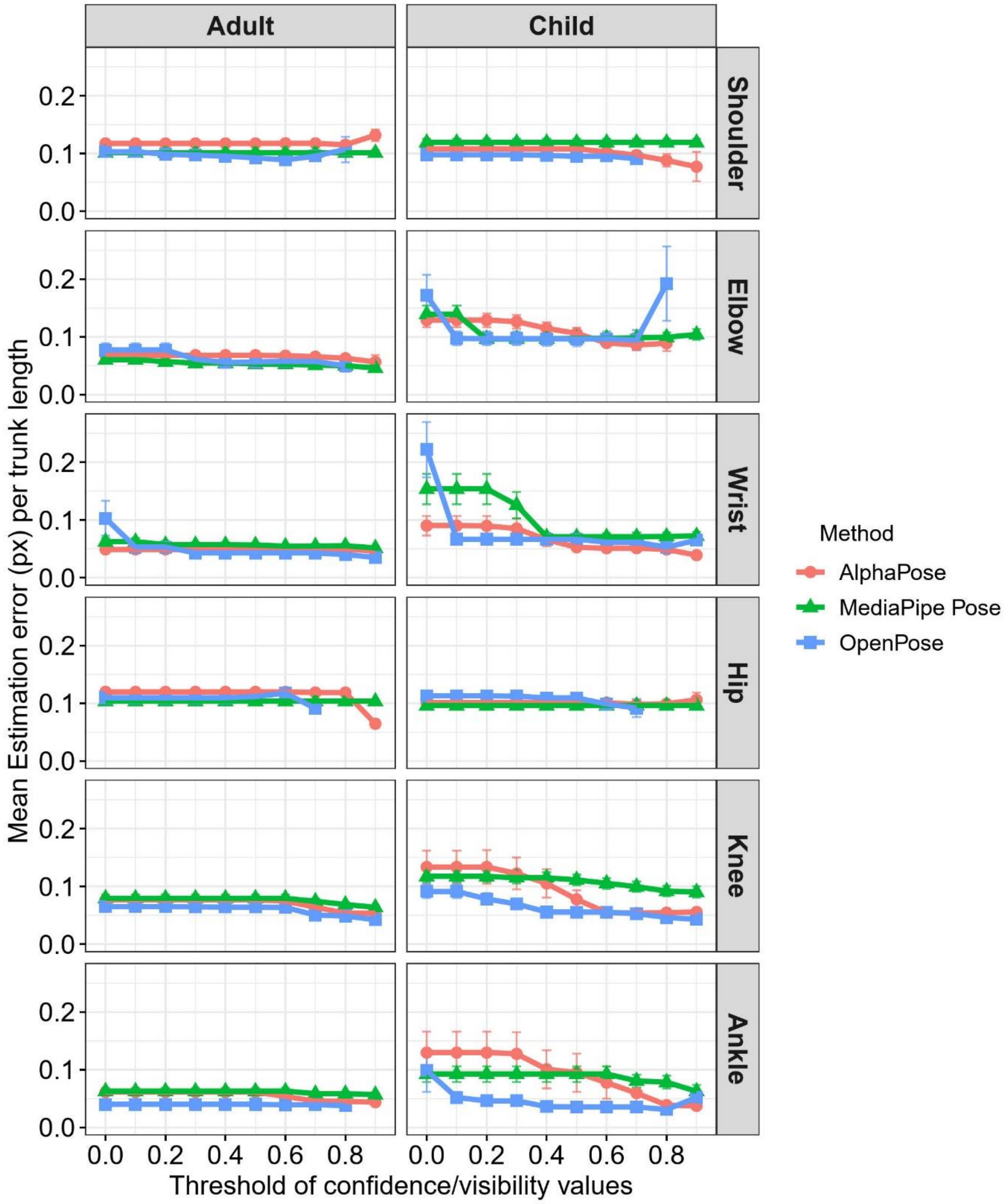

Figure S2. Estimation error distance of each pose estimation method for each keypoint.
Note. Error bars indicate standard error.

Table S1. List of abbreviations

AG	antigravity score
AIC	Akaike’s Information Criterion
EMA	exponentially weighted moving average
EWMSD	exponentially weighted moving standard deviation
FPS	frame per second
JPAN	Japanese Playful Assessment for Neuropsychological Abilities
OT	occupational therapist
SPB	static postural balance score
XAI	explainable artificial intelligence
